# Social capital predicts accelerometry-measured physical activity among older adults in the U.S.: a cross-sectional study in the National Social Life, Health, and Aging Project

**DOI:** 10.1186/s12889-018-5664-6

**Published:** 2018-06-27

**Authors:** Erin C. Ho, Louise Hawkley, William Dale, Linda Waite, Megan Huisingh-Scheetz

**Affiliations:** 1 Pritzker School of Medicine, The University of Chicago, 924 E 57th St, Chicago, IL 60637 USA; 2 NORC at the University of Chicago, 1155 East 60th St, 2nd Floor, Chicago, IL 60637 USA; 3 Department of Medicine, Section of Geriatrics and Palliative Medicine, University of Chicago Medicine, 5841 South Maryland Ave, MC 6098, Chicago, IL 60637 USA; 4 Department of Sociology, The University of Chicago, 1126 E 59th St, Chicago, IL 60637 USA

**Keywords:** Older adults, Accelerometry, Physical activity, Social capital, Social network, Social engagement

## Abstract

**Background:**

Older adults receive important health benefits from more robust social capital. Yet, the mechanisms behind these associations are not fully understood. Some evidence suggests that higher levels of social capital ultimately affect health through alterations in physical activity (PA), but most of this research has relied on self-reported levels of PA. The aim of this study was to determine whether components of social capital, including social network size and composition as well as the frequency of participation in various social and community activities, were associated with accelerometry-measured PA levels in a nationally representative sample of community-dwelling older adults (≥ 62 years).

**Methods:**

We conducted a cross-sectional analysis using data from the wrist accelerometry sub-study (*n* = 738) within Wave 2 of the National Social, Health, and Aging Project (NSHAP), a population-based longitudinal study that collects extensive survey data on the physical, cognitive, and social health of older adults. Participants’ physical activity was measured with a wrist accelerometer worn for 72 consecutive hours. We related seven, self-reported social relationship variables (network size, network proportion friends, and frequencies of socializing with friends and family, visiting with neighbors, attending organized group meetings, attending religious services, and volunteering) to accelerometer-measured PA (mean counts-per-minute) using multivariate linear regression analysis, while adjusting for potential confounders.

**Results:**

Larger social networks (*p* = 0.042), higher network proportion friends (*p* = 0.013), more frequent visiting with neighbors (*p* = 0.009), and more frequent attendance at organized group meetings (*p* = 0.035) were associated with higher PA levels after controlling for demographic and health covariates. Volunteering was significant prior to adjusting for covariates. No significant associations were found between frequencies of socializing with friends and relatives or attendance at religious services and PA.

**Conclusions:**

This study suggests social capital is significantly related to objectively measured PA levels among older adults, and that friendships as well as social participation in groups and with neighbors may be particularly pertinent to PA. These findings expand our understanding of and offer a potential mechanism linking social relationships and overall health among older adults. They also have implications for how we might motivate older adults to be more physically active.

**Electronic supplementary material:**

The online version of this article (10.1186/s12889-018-5664-6) contains supplementary material, which is available to authorized users.

## Background

It has been well established, both conceptually and empirically, that more social capital (i.e., the relationships between people and the potential resources represented by these relationships) later in life is linked to a wide range of better health outcomes important to older adults. Key measures of social capital include 1) social network structure (i.e., the size and composition of one’s social network) and 2) levels of social engagement, (i.e., participation in social and community activities) [1]. Seminal research published by Berkman and Syme in 1979 [2] showed that the extent of one’s social network predicted 9-year mortality in a prospective study of adults in Alameda County, California, with the most isolated group being over two-times as likely to die during the follow-up period than the most socially connected group. Follow up work by House, et al. in 1982 [3] using data from the Tecumseh Community Health Study added that, in addition to one’s social network, increased participation in various social and community activities also predicted lower mortality in men over a 9–12 year follow-up period. Since then, a myriad of older-adult focused studies examining social capital have linked social networks and social engagement to a variety of key physical and mental health benefits in older adults, including protection against dementia [4, 5] and disability [6], higher levels of life satisfaction and perceived quality of life [7, 8], better self-rated health [9], and overall lower mortality [10].

While the mechanisms behind these beneficial associations are not yet fully understood [11], a leading theory is that social networks alter social and interpersonal behaviors, like physical activity (PA) which, in turn, influence health [12]. Physical activity has well established beneficial effects, both immediate and long-term, on the health of older adults [13]. Immediate benefits include improved sleep, insulin sensitivity, and reduced blood pressure [13]. In the long term, activity lowers risk for coronary heart disease, diabetes, colon and breast cancers, fractures and falls, dementia, and depression and anxiety [14]. Physical activity promotes feelings of well-being and delays disability and frailty for older adults, both of which are crucial for maintaining independent living [15].

Previous studies have suggested some aspects of social capital may be related to physical activity, though few studies focus specifically on older adults and almost all use self-reported physical activity measures. In particular, social network composition, network size and social engagement may be related self-reported PA. Older adults reporting “diverse” or largely “friend”-based networks report higher PA levels than those who report primarily “family”-based or “restricted” networks [16–18]. In fact, Loprinzi, Joyner et al. in 2016 found a dose-related relationship such that the greater the number of close friends reported in an older adult’s network, the greater their self-reported physical activity participation [19]. Social engagement with friends and family, whether in formal or informal groups, has repeatedly been seen to predict self-reported physical activity levels in middle-aged and older adults [20–24]. Conversely, those with greater social isolation, defined by a lack of social network ties and social integration, are the least physically active [25, 26]. One significant drawback of the above studies is that they rely on self-reported PA measures, which are subject to recall bias [27] and tend to overestimate true PA levels when compared to physical activity measured by accelerometry [27, 28].

To the best of our knowledge, no studies have directly related objective PA levels to social network and social engagement measures in older adults. Loprinzi, Crush et al. in 2018 related social support network measures to accelerometry-measured sedentary behavior, rather than physical activity, and found spousal social support for men and larger household sizes to be associated with lower levels of sedentary behavior [29]. Carlson et al. reported an association between accelerometry-measured PA and social support related specifically to PA (such as friends or family walking with, exercising with, or encouraging the participant to exercise) but did not study other aspects of social capital such as social network size or composition or social engagement [30].

To extend the existing research, the aim of this study was to relate an objective measurement of physical activity, wrist accelerometry [28, 31], to social capital measures (social networks and social engagement) in a nationally representative dataset of older adults in the United States. We analyzed social capital measures pertaining to social network structure and levels of engagement in social and community activities, all of which are unique and independently important aspects of social health that are modifiable by physicians, caretakers, and policy makers through targeted public health initiatives. We hypothesized that older adults with larger network sizes, a friend-focused network composition, and higher levels of social engagement would exhibit higher accelerometer-measured physical activity levels.

## Methods

### Study design and sample

We conducted a cross-sectional analysis of community-dwelling U.S. older adults using data from the National Social Life, Health and Aging Project (NSHAP), a population-based longitudinal study that collects extensive survey and biomeasure data on the physical, cognitive, and social health of older adults [32]. The NSHAP data collection methods have been previously described in detail [33]. In brief, Wave 1 (2005–6) consisted of a nationally-representative probability sample of community-dwelling adults born between 1920 and 47 (aged 57–85), including oversamples of African-Americans, Hispanics, and men [33]. Five years later, respondents remaining alive were re-interviewed in Wave 2 (2010–2011) along with their cohabiting spouse or partner, for a total Wave 2 sample size of 3377. Respondents were interviewed in their homes by professional interviewers from NORC at the University of Chicago. By random selection, one third of the Wave 2 respondents were invited to participate in a sleep-activity module. The sleep-activity module included wearing an accelerometer as well as completing additional questionnaires [34, 35]. The NSHAP study was approved by NORC’s Institutional Review Board and secondary analysis of de-identified data was deemed exempt from further review by the University of Chicago Institutional Review Board (IRB# 17–0854). Our sample of interest consisted of a subset of 738 respondents (age range: 62–91) in Wave 2 randomly chosen to participate in the wrist accelerometer sub-study.

### Wrist accelerometer data collection and physical activity measures

The primary outcome of interest for this study was average daily physical activity, as assessed by wrist accelerometry. Participants in the accelerometer sub-study wore the Actiwatch Spectrum (Philips Respironics, Aurora, IL) [36] on their non-dominant wrist for 72 consecutive hours. The Actiwatch Spectrum is a validated, uniaxial, omnidirectional, and waterproof, piezoelectric device [37]. Detailed descriptions of the device and usage in NSHAP have been published elsewhere [34]. Briefly, the device continuously collects acceleration and deceleration data, which is then averaged over one-minute intervals (epochs) and recorded as an activity count. When no activity occurred over a given minute, such as during sleep or rest, the activity count was recorded as “0”. Non-wear time was automatically detected by a built-in galvanic heat sensor and excluded from analysis. Sleep and wake intervals were determined using manufacturer-suggested guidelines and were manually curated to account for respondent recordings in a take-home sleep log, accelerometer data on ambient light, and participant event markers noting time to bed and time awake [35]. Following best practices in accelerometry data analysis, only days with 10 h or more of wake time accelerometry recordings were considered valid days of activity measurement and included in analyses. This necessary requirement reduced the skewing of average activity calculations due to abbreviated wear times.

For this analysis, *mean daily counts* were calculated for each participant as an estimate of average daily activity volume by taking the total wake activity counts divided by the total wake hours worn. The number of hours worn, number of weekend days worn (0, 1, or 2), and month of wear (factor variable, reference = January) were also recorded and adjusted for in all analyses to adjust for variations in activity resulting from seasonality of the wear period and consistently observed differences between weekday and weekend physical activity levels among adults [34, 38].

### Social relationship characteristics

The social capital data collected in NSHAP covered two overarching dimensions: 1) social networks and 2) social engagement. Within social networks, we considered *network size* and *network proportion friends. Network size* refers to the number of individuals with whom the respondent feels comfortable discussing important matters [32]. Network size in NSHAP was determined using NSHAP’s network roster module, which asked respondents to name up to five people with whom the respondent had discussed important matters within the last 12 months [32]. The number of names that the respondent listed was summed to generate a network size (range 0–5), 0 indicating a network size of 0 persons. *Network proportion friends* was analyzed as the number of people named in the network as a friend divided by the total number of people named in the network (range 0–1).

The level of social engagement refers to the existence, quantity, and frequency of contact with particular informal and formal ties [11]. There were five social engagement questions included in NSHAP, four of which asked about the frequencies within the last year that the participant 1) socialized with friends or relatives, 2) attended religious services, 3) attended organized group meetings (e.g., a choir, a committee or board, a support group, a sports or exercise group, a hobby group, or a professional society), and 4) volunteered for religious, charitable, political, health-related, or other organizations. The fifth social engagement question asked about the frequency with which neighbors in the area visit in each other’s homes or on the street. Response options for all of the social engagement questions except ‘visiting with neighbors’ used a 7-point scale (never, less than once a year, about once or twice a year, several times a year, about once a month, every week, and several times a week). The frequency of ‘visiting with neighbors’ was a 4-point scale (never, rarely, sometimes, and often). Responses from all five questions were recoded during analysis to a condensed 3-point scale and treated as interval variables (never/rarely, sometimes, and often). Leveraging prior categorizations by Kotwal et al. [39], we also grouped the social engagement variables into two composite scales: *socializing* (calculated as the sum of the frequencies of socializing with friends / relatives and visiting with neighbors, range: 0–4) and *community involvement* (calculated as the sum of the frequencies of attending organized group meetings, attending religious services and volunteering, range: 0–6).

### Covariates

We included demographics, comorbidities, functional status, mental and cognitive status, and self-rated physical health status as covariates that were considered to be potential confounders in our analysis.

#### Demographics

Demographic covariates included participants’ self-reported *age* (continuous), *gender* (female versus male), *education* (< high school, high school/GED, some college, bachelor’s degree or higher), *marital status* (married/living with partner versus single), *employment status* (currently working versus not working) and *net worth* which was calculated as household net worth of property and financial assets after accounting for outstanding loans (<$10 k, $10-$49 k, $50-$99 k, $100-$499 k, $500 k+, unreported) (Table 1).

**Table 1:** Descriptive statistics (*n* = 673)

Variable	Mean (SD) or %
Physical Activity	
Mean Daily Counts (counts/minute)	222.9 (88.6)
Accelerometer Wear	
Mean Wear Time (hours)	36.3 (7.9)
Participants Wearing Accelerometer over 1 Weekend Day (%)	18.4%
Participants Wearing Accelerometer over 2 Weekend Days (%)	22.1%
Demographics	
Mean Age (years)	71.9 (7.2)
Female (%)	54%
Education (%)	
< High School	13.6%
High School/GED	24.8%
Some College/Vocational	37.7%
Bachelors or More	23.9%
Race/Ethnicity (%)	
White/Caucasian	83.9%
Black/African-American	6.4%
Hispanic (Non-Black)	6.2%
Other	3.5%
Married/Living with a Partner (%)	62.9%
Net Worth (%)	
< $10 k	5.6%
$10 k-$49 k	9.6%
$50 k-$99 k	10.1%
$100 k-$499 k	39.2%
$500 k+	27.2%
Unreported	8.3%
Currently Working (%)	24.8%
Comorbidities	
Modified Charlson Comorbidity Index^a^	0.98 (1.33)
Body Mass Index (%)	
Underweight or Normal (BMI 18–24)	25.3%
Overweight (BMI 25–30)	35.4%
Obese (BMI ≥30)	39.3%
Functional Health	
Difficulty Performing ≥1 ADL^b^ (%)	31%
Difficulty Performing ≥1 IADL^c^ (%)	38.9%
Slow Gait^d^ (%)	28.7%
Weakness^d^ (%)	28.5%
Mental and Cognitive Health	
Montreal Cognitive Assessment – Survey Adapted^e^	23.3 (3.9)
Modified CES-Depression Survey^f^	15.1 (3.9)
Self-Rated Health Status	
Poor/Fair Physical Health (%)	19.8%

Abbreviations: *SD* Standard deviation, *BMI* Body mass index, *ADL* Activities of daily living, *IADL* Instrumental activities of daily living, *CES* Center for epidemiological studies

^a^Modified Charlson Comorbidity Index (CCI) provides a summative level of comorbidities for each respondent. Survey modified CCI has a range 0–14 and scoring includes any heart condition, stroke, diabetes, cancer not including skin, metastatic cancer, emphysema, asthma, chronic bronchitis or COPD, Alzheimer’s disease, dementia

^b^Activities of Daily Living include walking one block, dressing, walking across a room, transferring in/out of bed, toileting, bathing, and eating

^c^Instrumental Activities of Daily Living include using the telephone, driving at night, driving during the day, light housework, shopping for food, meal preparation, managing money, and taking medications

^d^Slow Gait was defined as taking ≥5.7 s to complete a 3-m timed walk (the faster of two attempts used). Weakness was defined as taking ≥16.7 s to complete 5 repeated chair stands. Wheelchair-bound participants and those who could not complete the task safely were also considered ‘slow’and ‘weak’

^e^Montreal Cognitive Assessment (MoCA) values (range 0–30) were estimated from an 18-item survey adaptation of the MoCA developed for NSHAP Wave 2

^f^Modified Center for Epidemiological Studies-Depression survey, measured by 11 separate items with range of 0 to 33

#### Comorbidities

Weight status was assessed via calculation of *body mass index* (BMI) [40] using collected height and weight data, which was further categorized into underweight/normal (BMI 16 to < 25), overweight (BMI 25 to < 30), and obese (BMI ≥ 30). Level of comorbidities was assessed using a survey-modified *Charlson Comorbidity Index* (CCI) (range 0–14, continuous) [41].

#### Functional status

We controlled for degree of disability and frailty which may contribute independently to physical inactivity. Disability was identified when respondents reported having any *difficulty with one or more instrumental activities of daily living* (IADLs: using the telephone, driving at night, driving during the day, light housework, shopping for food, meal preparation, managing money, and taking medications) (versus no IADL difficulty) or having *difficulty with one or more activities of daily living* (ADLs: walking one block, dressing, walking across a room, transferring in/out of bed, toileting, bathing, and eating) (versus no ADL difficulty). We used *slow gait* and *weakness* to adjust for frailty. *Slow gait* was measured directly by asking respondents to walk 3 m at their “usual” pace; those requiring ≥5.7 s to complete the faster of two timed walks—as well as those who were wheelchair bound or could not complete the exercise safely—were determined to have slow gait [34]. To identify *weakness,* respondents were asked to stand up and sit-down from a chair five times as quickly as possible and were considered ‘weak’ if they required ≥16.7 s to complete the exercise, were wheelchair bound or could not complete the task safely [34]. Participants with missing data for these measures (*n* = 14 for timed gait, *n* = 30 for chair stands) were categorized separately and included in analyses.

#### Cognitive and mental status

*Depressive symptoms* were measured using an adaptation of the Center for Epidemiological Studies-Depression (CES-D) scale (range 0–33, continuous variable) [42]. *Cognitive status* was measured using an 18-item survey-adapted Montreal Cognitive Assessment (MoCA-SA), from which MoCA scores (range 0–30, continuous) were estimated using a linear prediction model [39].

#### Self-rated health status

*Self-rated physical health status* was reported as poor, fair, good, very good, or excellent.

### Statistical analysis

Descriptive statistics were generated for the study sample using means and frequencies. Correlations among all variable pairs were estimated using Spearman correlation coefficients. Multivariate linear regression models were used to explore how physical activity varied with each social capital variable. A first set of regression models were run for each social capital variable while controlling for wrist accelerometry wear time, number of weekend days worn, and wear month (minimally adjusted models). A second set of regression models additionally controlled for demographics, comorbidities, functional health, cognitive and mental health, and self-rated physical health (fully adjusted models). All models incorporated survey weights distributed with the dataset to make the estimates reflective of the U.S. population in 2010 by accounting for the differential probabilities of participant selection embedded in the survey design and nonresponse [33]. A *p*-value of < 0.05 was accepted as a statistically significant finding. Analyses were conducted using Stata version 14 (Stata Corp., College Station, TX).

## Results

Descriptive demographic, health and activity characteristics of the study sample are listed in Table 1. Of the 738 participants in the accelerometry sub-study, 673 had the complete set of accelerometry, demographic, and health data required to be included in the study sample. The mean age was 71.9 years (SD: 7.2 years), 54.0% were female, and 83.9% were White/Caucasian. A majority (62.9%) was married and 24.8% was still working. One in five participants rated their physical health as poor or fair. Some degree of disability was common in this sample: 31.0 and 38.9% of the sample reported having difficulty performing at least one ADL and at least one IADL, respectively.

The group wore the wrist accelerometers for an average of 36.3 waking hours (SD: 7.9 h) over the 72-h wear period; 18.4% wore the accelerometer on one weekend day and 22.1% wore the accelerometer over both weekend days. The mean accelerometer-measured activity level was 222.9 counts-per-minute (SD: 88.6 counts-per-minute).

Social relationship characteristics are described in Table 2. The mean network size was 3.9 persons (SD: 1.3 persons) and 47.6% of the sample reported having 5 people (the maximum number recorded) in their network. The mean network proportion consisting of friends was 0.27 (SD: 0.29). The reported frequencies of visiting with neighbors, attending a community group meeting, attending a religious service, and volunteering were relatively equally distributed across response options; however, 53.3% of respondents reported socializing with friends or relatives ‘often’ and 57.0% of respondents did not volunteer in the past year.

**Table 2:** Social relationship characteristics (*n* = 673 unless otherwise noted)

Variable	Mean (SD) or %
Network Structure	
Network Size^a^ (persons)	3.9 (1.3)
Network Size (%)	
0	0.3%
1	5.4%
2	9.7%
3	19%
4	17.9%
5	47.6%
Proportion ‘Friends’ in Network	0.27 (0.29)
Social Engagement Frequency	
Socializing with Friends/Relatives (*n*^b^=605) (%)	
Never/Rarely	8.6%
Sometimes	38.1%
Often	53.3%
Visiting with Neighbors (*n*^b^=613) (%)	
Never/Rarely	38.6%
Sometimes	39%
Often	22.4%
Attending Community or Group Meetings (*n*^b^=606) (%)	
Never/Rarely	45.6%
Sometimes	24.8%
Often	29.6%
Attending Religious Services (*n*^b^=670) (%)	
Never/Rarely	38.3%
Sometimes	16.2%
Often	45.5%
Volunteering (*n*^b^=604) (%)	
Never/Rarely	57%
Sometimes	18%
Often	25%

^a^Network size (range 0 to 5) refers to the number of persons with whom the respondent “discussed important matters” in the past 12 months

^b^Participants with non-missing data for accelerometry measures, covariates, and the social variable

Multivariate linear regression models (Table 3 and Additional file 1), each consisting of *mean daily counts* as the outcome variable and a single social capital variable (e.g., *network size*) plus covariates as the independent variables, revealed that a number of social capital measures were significantly associated with accelerometry-assessed activity. When adjusting for accelerometer-wear covariates only (Table 3: Minimally Adjusted Regression Models adjusted for accelerometer number of hours worn, number of weekend days worn, and month of wear), all hypothesized social capital measures were significantly associated with physical activity: larger network size (*β* = 6.16, *p = 0.033*)*,* higher network proportion friends (*β* = 36.91, *p = 0.029*), more frequent socializing (*β* = 12.35, *p = 0.006*) and higher levels of community involvement (*β* = 4.5 *p = 0.033*) all predicted higher PA levels. After additionally controlling for demographic, health, and accelerometry covariates (Table 3: Fully Adjusted Regression Models), network size (*β* = 4.77, *p = 0.042),* network proportion friends (*β* = 35.81, *p = 0.013)* and frequency of socializing (*β* = 8.73, *p = 0.028)* persisted as significant predictors of activity.

**Table 3:** Multivariate linear regression: social network and social engagement predictors of wrist accelerometer-measured physical activity levels

				Minimally Adjusted Regression Models^a^		Fully Adjusted Regression Models^b^	
Model	Independent Variable		*n*	β Coefficient (CI)	*p* value	β Coefficient (CI)	*p* value
Social Network Structure							
1	Network Size		662	6.16 (0.51–11.82)	0.033	4.77 (0.18–9.35)	0.042
2	Network Proportion Friends		662	36.91 (3.9–69.91)	0.029	35.81 (7.97–63.66)	0.013
Social Engagement Frequency							
3	Socializing^c^		596	12.35 (3.73–20.97)	0.006	8.73 (1–16.46)	0.028
	3a	Socializing with Friends/Relatives^d^	596	12.79 (− 2.33–27.9)	0.095	5.72 (− 6.35–17.8)	0.35
	3b	Visiting Neighbors^e^	604	17.87 (7.04–28.69)	0.002	14.62 (3.86–25.37)	0.009
4	Community Involvement^f^		590	4.5 (0.38–8.63)	0.033	3.33 (− 0.35–7.02)	0.075
	4a	Attending Organized Group Meetings^d^	597	11 (1.84–20.17)	0.020	9.04 (0.66–17.41)	0.035
	4b	Attending Religious Services^d^	659	3.59 (− 6.4–13.58)	0.47	3.57 (− 4.37–11.51)	0.37
	4c	Volunteering^d^	595	10.39 (0.45–20.32)	0.041	5.97 (− 3.53–15.47)	0.21

^a^Each minimally adjusted linear regression model is controlled for accelerometer number of hours worn, number of weekend days worn, and month of wear

^b^Each fully adjusted linear regression model is adjusted for age, gender, education, ethnic group, net worth, marital status, job status, self-rated health, functional health, comorbidities, depressive symptoms, and cognitive status in addition to accelerometer number of hours worn, number of weekend days worn, and month of wear

^c^Socializing refers to the composite scale comprised of frequencies of socializing with friends/relatives and visiting with neighbors

^d^Frequency with which respondent participates in the specified social engagement activity in the past 12 months

^e^Frequency with which respondent or his/her neighbors visit with one another in homes or on the streets

^f^Community involvement refers to the composite scale comprised of frequencies of attending organized group meetings, attending religious services, and volunteering

**Fig. 1:**
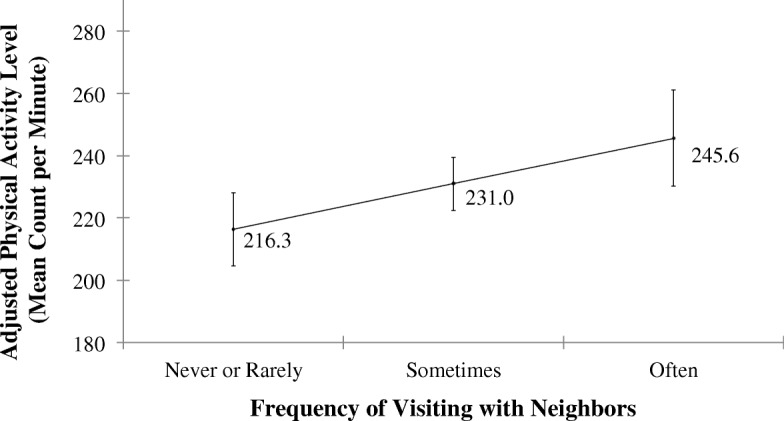
Adjusted predictions of mean PA levels as a function of frequency of visiting with neighbors. Adjusted marginal estimates of the association between ‘physical activity level’ and ‘frequency with which respondent or neighbors in the area visit one another’ after controlling for all covariates (Multivariate Linear Regression Model #3b). Marginal estimates adjusted for age, gender, education, ethnic group, net worth, marital status, self-rated health, functional health, comorbidities, depressive symptoms, cognitive status, and accelerometer wear time/day. Bars represent 95% confidence intervals

Additional regression analyses were conducted on the individual social activities comprising the composite social engagement measures to assess whether the effects of certain activities predominated in their associations with physical activity. For socializing (Table 3: model 3), the frequency of visiting with neighbors (Table 3: model 3b, fully adjusted, *β* = 14.62, *p* = 0.009) was found to largely account for the predictive value of socializing as a composite measure. To illustrate, after adjusting for all other covariates, older adults who reported ‘often’ visiting with neighbors had a predicted mean activity level that was 13.5% higher than those who reported ‘never or rarely’ visiting with their neighbors, 245.6 cpm (95% CI: 230.2–261.0) vs. 216.3 cpm (95% CI: 204.6–228.0) (Fig. 1). In contrast, the frequency of socializing with friends/relatives (Table 3: model 3a) did not significantly predict physical activity, even prior to full adjustment with covariates. For community involvement (Table 3: model 4), attendance at organized group meetings showed a positive association with PA that persisted after adjustment for demographic and health covariates (Table 3: model 4a, fully-adjusted, *β* = 9.04, *p* = 0.035). Frequency of volunteering was significantly associated with PA when adjusting for accelerometry covariates only (Table 3: model 4c, minimally adjusted, *β* = 10.39, *p* = 0.041) and lost significance as a predictor of physical activity after controlling for demographic variables, notably gender and assets. No significant relationship was found between physical activity and frequency of attending religious services (Table 3: model 4b).

## Discussion

We are among the first to use an objective measure of physical activity to explore how social capital is related to physical activity in a nationally representative sample of U.S. older adults. We found significant associations between social capital and accelerometry-measured physical activity, even after adjusting for potential confounders. Most notably, larger-sized networks, networks with higher proportion of friends, and more frequent socialization with neighbors predicted higher PA levels.

The positive association between total network size and physical activity agreed with findings from previous research studies using self-reported physical activity data [2, 16, 18, 19]. One potential explanation is that larger social networks protect against the detrimental psychological and physiological effects of social isolation [2, 25]. The network size captured by NSHAP, which includes people that the respondent feels close enough with to discuss important matters, indicates the extent to which the respondent has strong, frequently accessed relationships through which support and resources can flow [32]. Conversely, respondents in NSHAP with small to nonexistent network are likely to feel socially isolated, a major risk factor for and/or consequence of various negative health behaviors and outcomes, including decreased physical activity levels and increased sedentary time [34, 35].

We also found that study participants reporting networks with higher proportions of friends also had higher levels of accelerometry-measured PA. Prior research characterizing friendships in older adults has found that interactions with friends are more likely to be dictated by pleasure and enjoyment whereas family interactions often involve daily needs and routine tasks. Moreover, older adults frequently name friends as the network members with whom they enjoy spending their time and engaging in leisure activities [43], which substantiate our findings that older adults with relatively more friend-focused networks also had higher PA levels. Our findings add to the growing evidence that maintaining friendships later in life may benefit health and/or reflect better health in ways that having relationships with family members cannot [19, 43–46].

We found the predictive value of frequency of *socializing* seen in our study to be driven by a strong association between visiting with neighbors and physical activity. Our analyses showed that, all else being equal, an older adult who often visited with their neighbors was predicted to have a daily physical activity level that was 13.5% higher than an older adult who never or rarely visited with their neighbors (Fig. 1). Recent studies have concluded that even small increases in activity level (or decreases in sedentary time) are associated with meaningful health benefits [47, 48]; likewise, the 13.5% increase associated with visiting neighbors seen in this study suggests a clinically meaningful increase in activity, especially for older adults who spend the majority of their time completely inactive. Moreover, due to the wording of this question (“How often do you and people in this area visit in each other’s homes or when you meet on the street?”), responses may have reflected characteristics of the respondent’s neighborhood and built environment as much as the respondent’s own social behaviors and preferences. For instance, Van Holle et al. [20] found that higher frequencies of talking with neighbors were related to higher levels of self-reported walking for recreation or transport in a sample of community-dwelling Belgian older adults. Our finding, along with other studies observing the impact of neighborhood walkability, aesthetics, accessibility to parks, and neighborhood safety on self-reported physical activity levels [20, 49–51], points to the potential physical and social health importance of the neighborhood and environment in which an older adult lives.

In addition to visiting with neighbors, two of the *community involvement* activities, attendance at organized group meetings and volunteering, significantly predicted PA levels. The relationship between organized group meeting attendance and PA was not surprising, as one of the examples of organized group meetings listed in the survey questionnaire was “a sports or exercise group.” Still, the observed relationship is useful in testing the validity of our analysis, insofar as we did observe the existence of a significant relationship as expected. It also points to the effectiveness of participation in organized group exercise, among other group activities, as a way to engage older adults in physical activity. The association between volunteering frequency and PA, another relationship we expected, was attenuated by demographic variables. In particular, being female and/or having more assets predicted higher levels of both volunteering frequency and PA, and helped to explain the association between volunteering and PA, possibly indicating volunteering is a surrogate for these demographic measures. Longitudinal data are necessary to test whether changes in volunteering frequency predict changes in PA even after adjustment for demographic variables.

In contrast, frequencies of socializing with friends/relatives and religious service attendance were not related to physical activity levels. This may in part be explained by the nature of the activities themselves and whether they inherently require some minimum level of physical activity to participate. For example, compared to “get togethers” with friends and family that often occur seated inside an older adult’s home or a religious service during which participants are seated for the majority of the service duration, visiting with neighbors in each other’s homes or on the streets, participating in an exercise group, or volunteering generally require more physical activity. This suggests that not all social activities are equal in their relevance to PA. Future research should seek to replicate our findings and examine other specific types of social activities that may be relevant for PA and could be targets for interventions.

Our study has several limitations worth noting. This was a cross-sectional analysis and, therefore, we were only able to test for associations. It should be noted, though, that study participants were surveyed about their social capital prior to participating in the accelerometry sub-study. As longitudinal data become available in NSHAP, it will be possible to test causal models linking changes in social activity with subsequent changes in physical activity. Missing social data reduced our sample size, but the application of survey weights ensured the sample analyzed remained nationally representative, which is one of the notable strengths of this study [33, 52–54]. Finally, the accelerometer used in this study has several limitations. The hip has traditionally been the preferred location for activity monitoring and activity intensity assessment [55]. The NSHAP used a wrist worn activity device to maximize adherence and to simultaneously evaluate sleep [35]. Prior studies have shown that output from the Actiwatch Spectrum is strongly associated with important health indicators in older adults, such as disability [56, 57], supporting its utility as a measure of activity. The Actiwatch Spectrum does not have established cut-points distinguishing different intensities of activity as other accelerometers have [58]. However, accelerometer count cut-points have variable accuracy in predicting energy expenditure among older adults [59]. The variable accuracy may be due, in part, to common age-related conditions that slow speed yet increase energy costs of mobility such as gait impairment [60]. We alternatively used mean daily activity counts to give an estimate of average activity volume. The short wear time protocol (72 h) is not ideal; however, prior work has found average daily activity assessed over 2 days versus 7 days is highly correlated (Lin’s concordance correlation coefficient > 0.9) among U.S. older adults [38]. Furthermore, the shortened wear time protocol facilitates adherence allowing inclusion of a more representative sample of older adults [38]. Despite these limitations, our use of wrist accelerometry extends prior work utilizing self-reported PA data. Wrist accelerometry is being deployed in each successive wave of NSHAP data to facilitate longitudinal analyses involving PA in older American adults.

## Conclusions

This study extends prior research finding an association between aspects of social capital and self-reported physical activity by demonstrating associations between specific aspects of social capital and an objective accelerometry-based measure of physical activity. Although the causal direction of these associations remains to be determined, this study highlights a need to emphasize the social dimension of older adult health in the medical and public health systems to consider how social capital can be promoted to improve older adult health. Only 6–20% of older adults over 60 years of age reach the recommended activity targets [58, 61], and most spend the large majority of their waking hours completely sedentary [62, 63]. Social health interventions on an individual or community level could be used to motivate older adults to become more physically active. Intervention efforts will benefit from future work that explores the mechanisms by which social capital may promote physical activity among older adults.

## Additional file

Additional file 1: Contains the complete results of the minimally adjusted and fully adjusted multivariate regression models for each of the four major models highlighted in Table 3 (models 1, 2, 3 and 4).ᅟ(DOCX 127 kb)

## Abbreviations

ADL: Activities of daily living
BMI: Body mass index
CES: Center for Epidemiological Studies
CI: Confidence interval
IADL: Instrumental activities of daily living
MoCA: Montreal Cognitive Assessment
MoCA-SA: Montreal Cognitive Assessment-Survey Adapted
PA: Physical activity
SD: Standard deviation
