# Supplementary material for: Social capital predicts accelerometry-measured physical activity among older adults in the U.S.: a cross-sectional study in the National Social Life, Health, and Aging Project
Source: BMC Public Health. 2018 Jun 27;18:804. doi: 10.1186/s12889-018-5664-6 (PMC6020417; doi:10.1186/s12889-018-5664-6)
Supplement: Supplementary file 1 — Contains the complete results of the minimally adjusted and fully adjusted multivariate regression models for each of the four major models highlighted in Table 3 (models 1, 2, 3 and 4).ᅟ(DOCX 127 kb) [file 12889_2018_5664_MOESM1_ESM.docx]

**Model #1: Social Network Size – Minimally Adjusted**

Survey: Linear regression

Number of strata = 49 Number of obs = 3,361

Number of PSUs = 98 Population size = 3,362.3439

Subpop. no. obs = 672

Subpop. size = 674.076717

Design df = 49

F( 15, 35) = 54.47

Prob > F = 0.0000

R-squared = 0.0735

------------------------------------------------------------------------------

| Linearized

mean_daily~m | Coef. Std. Err. t P>|t| [95% Conf. Interval]

-------------+----------------------------------------------------------------

netsizeAw2 | 6.162284 2.813265 2.19 0.033 .5088162 11.81575

|

day1_mon |

2 | 19.62283 27.13775 0.72 0.473 -34.91253 74.15818

3 | -29.24336 21.49167 -1.36 0.180 -72.43249 13.94577

4 | 25.66775 21.73407 1.18 0.243 -18.0085 69.34399

5 | -.9029539 19.55604 -0.05 0.963 -40.20229 38.39639

6 | 33.35133 29.19335 1.14 0.259 -25.31489 92.01756

8 | -112.1407 18.5197 -6.06 0.000 -149.3574 -74.92396

9 | -1.591389 19.57268 -0.08 0.936 -40.92416 37.74138

10 | 4.665483 16.10689 0.29 0.773 -27.70253 37.0335

11 | 10.40548 18.18986 0.57 0.570 -26.14841 46.95937

12 | 10.98868 19.6872 0.56 0.579 -28.57422 50.55159

999 | 230.7269 169.7873 1.36 0.180 -110.4735 571.9274

|

weekend_sum |

1 | -16.46504 7.960598 -2.07 0.044 -32.46246 -.4676229

2 | -18.26836 9.311233 -1.96 0.055 -36.97999 .4432591

|

n_total_cpm | .004566 .0076137 0.60 0.551 -.0107344 .0198664

_cons | 188.0876 31.16997 6.03 0.000 125.4492 250.726

------------------------------------------------------------------------------

Note: 1 stratum omitted because it contains no subpopulation members.

(running regress on estimation sample)

**Model #1: Social Network Size – Fully Adjusted**

Survey: Linear regression

Number of strata = 49 Number of obs = 3,351

Number of PSUs = 98 Population size = 3,354.9365

Subpop. no. obs = 662

Subpop. size = 666.669332

Design df = 49

F( 45, 5) = 96.64

Prob > F = 0.0000

R-squared = 0.3176

-----------------------------------------------------------------------------------

| Linearized

mean_daily_cpm | Coef. Std. Err. t P>|t| [95% Conf. Interval]

------------------+----------------------------------------------------------------

netsizeAw2 | 4.76888 2.281836 2.09 0.042 .1833584 9.354402

|

day1_mon |

2 | 17.50744 24.45735 0.72 0.477 -31.64144 66.65632

3 | -19.72071 18.87916 -1.04 0.301 -57.6598 18.21837

4 | 18.28641 22.4677 0.81 0.420 -26.86412 63.43694

5 | 12.29844 22.93603 0.54 0.594 -33.79324 58.39012

6 | 17.53651 24.76044 0.71 0.482 -32.22146 67.29448

8 | -94.56015 28.33889 -3.34 0.002 -151.5093 -37.61102

9 | 20.35106 16.19631 1.26 0.215 -12.19665 52.89876

10 | 14.50401 14.12958 1.03 0.310 -13.89045 42.89848

11 | 15.4926 15.95276 0.97 0.336 -16.56568 47.55087

12 | 9.729922 16.26483 0.60 0.552 -22.95548 42.41532

999 | 227.4259 152.157 1.49 0.141 -78.34508 533.1968

|

weekend_sum |

1 | -8.230229 7.96809 -1.03 0.307 -24.2427 7.782247

2 | -13.63229 7.214893 -1.89 0.065 -28.13116 .8665767

|

n_total_cpm | .0054212 .006312 0.86 0.395 -.0072632 .0181056

age | -2.875619 .6291023 -4.57 0.000 -4.139847 -1.611391

|

gender |

2. female | 38.39173 7.529469 5.10 0.000 23.2607 53.52277

|

ethgrp |

2. black | -6.964476 12.25805 -0.57 0.573 -31.59795 17.66899

3. hispanic, n.. | 24.92727 13.33589 1.87 0.068 -1.872205 51.72674

4. other | 13.49915 14.97789 0.90 0.372 -16.60005 43.59835

|

educ |

2. hs/equiv | 16.32617 12.65447 1.29 0.203 -9.103942 41.75628

3. voc cert/so.. | 5.608348 11.94835 0.47 0.641 -18.40276 29.61946

4. bachelors o.. | -2.136079 15.90946 -0.13 0.894 -34.10734 29.83518

|

married |

yes | 7.583942 7.235153 1.05 0.300 -6.955642 22.12353

|

jobstat_1 |

1. yes | 20.58173 8.74903 2.35 0.023 2.999896 38.16356

|

assets2 |

10k-49k | 9.660215 17.20028 0.56 0.577 -24.90504 44.22547

50k-99k | 20.94097 19.00969 1.10 0.276 -17.26044 59.14238

100k-499k | 31.12659 15.96258 1.95 0.057 -.9514101 63.20459

500k+ | 31.38731 15.8839 1.98 0.054 -.5325844 63.30721

missing | 50.19763 19.3954 2.59 0.013 11.22112 89.17415

|

ibmi |

2. overwt | -17.7201 9.46824 -1.87 0.067 -36.74724 1.307044

3. obese | -40.70211 9.53863 -4.27 0.000 -59.87071 -21.53352

|

charlsonmod | -5.043106 2.603036 -1.94 0.058 -10.2741 .1878902

|

new_adlcat |

at least 1 ADL.. | -3.329107 8.328962 -0.40 0.691 -20.06678 13.40857

|

new_iadlcat |

at least 1 IAD.. | 1.720712 8.545332 0.20 0.841 -15.45178 18.8932

missing | -1.752459 12.75724 -0.14 0.891 -27.3891 23.88418

|

new_slowgait2 |

1 | -11.11832 8.731147 -1.27 0.209 -28.66421 6.42758

missing | 12.23899 26.5411 0.46 0.647 -41.09735 65.57532

|

new_posweak2 |

1 | -14.92164 6.113881 -2.44 0.018 -27.20794 -2.635332

missing | 2.920936 14.67084 0.20 0.843 -26.56123 32.4031

|

moca | -1.375544 1.216892 -1.13 0.264 -3.820979 1.069891

cesd | .0559382 .8961066 0.06 0.950 -1.744855 1.856732

|

new_physhlth |

good | 4.243915 9.422066 0.45 0.654 -14.69044 23.17826

very good | 19.2756 9.519039 2.02 0.048 .1463709 38.40482

excellent | 12.90285 13.73366 0.94 0.352 -14.69597 40.50167

|

_cons | 381.1823 70.18207 5.43 0.000 240.1462 522.2185

-----------------------------------------------------------------------------------

Note: 1 stratum omitted because it contains no subpopulation members.

**Model #2: Network Proportion Friends – Minimally Adjusted**

Survey: Linear regression

Number of strata = 49 Number of obs = 3,361

Number of PSUs = 98 Population size = 3,362.3439

Subpop. no. obs = 672

Subpop. size = 674.076717

Design df = 49

F( 15, 35) = 54.62

Prob > F = 0.0000

R-squared = 0.0801

------------------------------------------------------------------------------

| Linearized

mean_daily~m | Coef. Std. Err. t P>|t| [95% Conf. Interval]

-------------+----------------------------------------------------------------

friendpcAw2 | 36.90636 16.42441 2.25 0.029 3.900272 69.91245

|

day1_mon |

2 | 26.58942 26.48662 1.00 0.320 -26.63744 79.81629

3 | -27.62306 20.99813 -1.32 0.194 -69.82038 14.57425

4 | 25.06795 21.76364 1.15 0.255 -18.66773 68.80363

5 | 2.872856 19.90532 0.14 0.886 -37.12839 42.8741

6 | 34.40267 31.87945 1.08 0.286 -29.66148 98.46683

8 | -116.6103 17.88342 -6.52 0.000 -152.5484 -80.6722

9 | 4.992235 20.27838 0.25 0.807 -35.75869 45.74316

10 | 7.087554 16.12049 0.44 0.662 -25.30779 39.4829

11 | 13.98923 18.39357 0.76 0.451 -22.97404 50.95249

12 | 15.64304 19.70075 0.79 0.431 -23.94711 55.23319

999 | 240.1733 167.9721 1.43 0.159 -97.37929 577.7259

|

weekend_sum |

1 | -17.94999 7.585035 -2.37 0.022 -33.19268 -2.70729

2 | -19.82953 9.354824 -2.12 0.039 -38.62876 -1.030313

|

n_total_cpm | .0074384 .0079205 0.94 0.352 -.0084785 .0233552

_cons | 193.5745 28.44653 6.80 0.000 136.4091 250.74

------------------------------------------------------------------------------

Note: 1 stratum omitted because it contains no subpopulation members.

(running regress on estimation sample)

**Model #2:** **Network Proportion Friends – Fully Adjusted**

Survey: Linear regression

Number of strata = 49 Number of obs = 3,351

Number of PSUs = 98 Population size = 3,354.9365

Subpop. no. obs = 662

Subpop. size = 666.669332

Design df = 49

F( 45, 5) = 83.91

Prob > F = 0.0000

R-squared = 0.3258

-----------------------------------------------------------------------------------

| Linearized

mean_daily_cpm | Coef. Std. Err. t P>|t| [95% Conf. Interval]

------------------+----------------------------------------------------------------

friendpcAw2 | 35.81164 13.85679 2.58 0.013 7.965366 63.6579

|

day1_mon |

2 | 24.17108 23.81 1.02 0.315 -23.67691 72.01908

3 | -16.42122 19.02019 -0.86 0.392 -54.64371 21.80128

4 | 18.57407 22.43392 0.83 0.412 -26.50859 63.65672

5 | 16.08825 23.4919 0.68 0.497 -31.12049 63.29699

6 | 20.7499 25.96127 0.80 0.428 -31.42122 72.92102

8 | -93.51538 28.30376 -3.30 0.002 -150.3939 -36.63685

9 | 27.26818 16.36214 1.67 0.102 -5.612776 60.14913

10 | 17.92064 13.86223 1.29 0.202 -9.936558 45.77785

11 | 19.35725 16.4126 1.18 0.244 -13.6251 52.33961

12 | 14.27064 16.42332 0.87 0.389 -18.73325 47.27453

999 | 237.0861 150.5316 1.57 0.122 -65.41849 539.5907

|

weekend_sum |

1 | -9.18851 7.665003 -1.20 0.236 -24.59191 6.214891

2 | -15.19019 7.005641 -2.17 0.035 -29.26855 -1.111823

|

n_total_cpm | .0078635 .006496 1.21 0.232 -.0051906 .0209176

age | -2.920802 .6519765 -4.48 0.000 -4.230998 -1.610607

|

gender |

2. female | 38.16311 7.534271 5.07 0.000 23.02243 53.30379

|

ethgrp |

2. black | -10.63507 11.94515 -0.89 0.378 -34.63975 13.36962

3. hispanic, n.. | 25.27664 13.29851 1.90 0.063 -1.447725 52.00101

4. other | 19.21682 16.44336 1.17 0.248 -13.82735 52.26098

|

educ |

2. hs/equiv | 14.17221 12.65592 1.12 0.268 -11.26083 39.60524

3. voc cert/so.. | 3.56224 11.34914 0.31 0.755 -19.2447 26.36918

4. bachelors o.. | -6.202596 15.8872 -0.39 0.698 -38.12913 25.72394

|

married |

yes | 9.599649 7.06037 1.36 0.180 -4.588696 23.78799

|

jobstat_1 |

1. yes | 20.00095 8.41632 2.38 0.021 3.087721 36.91418

|

assets2 |

10k-49k | 9.52353 17.40252 0.55 0.587 -25.44814 44.4952

50k-99k | 20.55468 18.9595 1.08 0.284 -17.54585 58.65521

100k-499k | 32.98076 16.20893 2.03 0.047 .4077011 65.55382

500k+ | 33.25385 16.51092 2.01 0.050 .0739058 66.43379

missing | 50.66364 19.3821 2.61 0.012 11.71385 89.61343

|

ibmi |

2. overwt | -17.2269 9.289835 -1.85 0.070 -35.89552 1.441726

3. obese | -40.67165 9.196756 -4.42 0.000 -59.15322 -22.19008

|

charlsonmod | -4.578249 2.553172 -1.79 0.079 -9.709041 .5525433

|

new_adlcat |

at least 1 ADL.. | -3.025542 8.143994 -0.37 0.712 -19.39151 13.34043

|

new_iadlcat |

at least 1 IAD.. | 2.524984 8.029836 0.31 0.755 -13.61157 18.66154

missing | -2.433531 11.7929 -0.21 0.837 -26.13226 21.26519

|

new_slowgait2 |

1 | -11.16173 8.771668 -1.27 0.209 -28.78905 6.465599

missing | 10.30174 27.74996 0.37 0.712 -45.4639 66.06739

|

new_posweak2 |

1 | -14.80207 5.909201 -2.50 0.016 -26.67705 -2.927084

missing | 2.462597 14.09291 0.17 0.862 -25.85816 30.78336

|

moca | -1.321626 1.156039 -1.14 0.258 -3.644774 1.001522

cesd | .0445816 .9252547 0.05 0.962 -1.814787 1.90395

|

new_physhlth |

good | 4.568357 9.498241 0.48 0.633 -14.51907 23.65579

very good | 18.80938 9.579842 1.96 0.055 -.4420325 38.0608

excellent | 12.99106 13.46667 0.96 0.339 -14.07123 40.05335

|

_cons | 383.1366 72.18818 5.31 0.000 238.069 528.2041

-----------------------------------------------------------------------------------

Note: 1 stratum omitted because it contains no subpopulation members.

**Model #3: Socializing - Minimally Adjusted**

Survey: Linear regression

Number of strata = 49 Number of obs = 3,361

Number of PSUs = 98 Population size = 3,362.3439

Subpop. no. obs = 604

Subpop. size = 614.766764

Design df = 49

F( 15, 35) = 48.11

Prob > F = 0.0000

R-squared = 0.1002

---------------------------------------------------------------------------------

| Linearized

mean_daily_cpm | Coef. Std. Err. t P>|t| [95% Conf. Interval]

----------------+----------------------------------------------------------------

cat3socializing | 12.34961 4.290261 2.88 0.006 3.728006 20.97121

|

day1_mon |

2 | 23.45547 27.03758 0.87 0.390 -30.87857 77.78952

3 | -27.58199 22.28517 -1.24 0.222 -72.36573 17.20174

4 | 25.87341 22.64059 1.14 0.259 -19.62457 71.37138

5 | 2.548564 20.48414 0.12 0.901 -38.61586 43.71299

6 | 40.1469 44.72908 0.90 0.374 -49.73955 130.0333

8 | -109.9465 18.88355 -5.82 0.000 -147.8944 -71.99861

9 | .1646077 22.32145 0.01 0.994 -44.69202 45.02124

10 | 6.180476 17.23118 0.36 0.721 -28.44687 40.80782

11 | 11.31998 19.33566 0.59 0.561 -27.53649 50.17645

12 | 21.53576 21.71921 0.99 0.326 -22.11063 65.18214

999 | 279.9064 213.2693 1.31 0.195 -148.6742 708.4871

|

weekend_sum |

1 | -15.31119 9.29082 -1.65 0.106 -33.98179 3.359414

2 | -20.70533 9.015132 -2.30 0.026 -38.82192 -2.588748

|

n_total_cpm | .0095006 .0090396 1.05 0.298 -.0086651 .0276663

_cons | 170.8036 30.45343 5.61 0.000 109.6052 232.0021

---------------------------------------------------------------------------------

Note: 1 stratum omitted because it contains no subpopulation members.

(running regress on estimation sample)

**Model #3: Socializing – Fully Adjusted**

Survey: Linear regression

Number of strata = 49 Number of obs = 3,353

Number of PSUs = 98 Population size = 3,355.8587

Subpop. no. obs = 596

Subpop. size = 608.28158

Design df = 49

F( 45, 5) = 33.14

Prob > F = 0.0005

R-squared = 0.3397

-----------------------------------------------------------------------------------

| Linearized

mean_daily_cpm | Coef. Std. Err. t P>|t| [95% Conf. Interval]

------------------+----------------------------------------------------------------

cat3socializing | 8.730856 3.844842 2.27 0.028 1.004356 16.45736

|

day1_mon |

2 | 23.73221 25.58483 0.93 0.358 -27.68243 75.14685

3 | -15.0502 18.26223 -0.82 0.414 -51.74951 21.64912

4 | 24.99961 22.67375 1.10 0.276 -20.56499 70.56422

5 | 18.68143 22.3446 0.84 0.407 -26.22172 63.58458

6 | 35.98087 29.86257 1.20 0.234 -24.03021 95.99196

8 | -89.05486 29.02399 -3.07 0.004 -147.3807 -30.72897

9 | 27.63324 15.51167 1.78 0.081 -3.538635 58.80511

10 | 21.01595 13.70254 1.53 0.132 -6.520334 48.55223

11 | 20.39714 17.42054 1.17 0.247 -14.61074 55.40502

12 | 24.58031 17.00698 1.45 0.155 -9.596503 58.75713

999 | 276.8486 186.0904 1.49 0.143 -97.11412 650.8112

|

weekend_sum |

1 | -8.079398 8.938368 -0.90 0.370 -26.04172 9.882925

2 | -15.03085 7.509282 -2.00 0.051 -30.12132 .0596142

|

n_total_cpm | .0106836 .0074075 1.44 0.156 -.0042023 .0255694

age | -2.876061 .5968097 -4.82 0.000 -4.075396 -1.676727

|

gender |

2. female | 41.32833 8.125831 5.09 0.000 24.99886 57.6578

|

ethgrp |

2. black | -13.15117 13.41569 -0.98 0.332 -40.11101 13.80868

3. hispanic, n.. | 22.47696 14.95729 1.50 0.139 -7.58084 52.53475

4. other | 16.14549 14.39967 1.12 0.268 -12.79174 45.08271

|

educ |

2. hs/equiv | 13.86903 13.13298 1.06 0.296 -12.52268 40.26073

3. voc cert/so.. | 2.36145 12.85805 0.18 0.855 -23.47777 28.20067

4. bachelors o.. | -5.341539 17.97112 -0.30 0.768 -41.45586 30.77278

|

married |

yes | 8.545965 7.435782 1.15 0.256 -6.396798 23.48873

|

jobstat_1 |

1. yes | 19.12452 9.658266 1.98 0.053 -.284495 38.53353

|

assets2 |

10k-49k | 13.4672 18.02609 0.75 0.459 -22.75759 49.69198

50k-99k | 29.05113 20.44912 1.42 0.162 -12.04292 70.14518

100k-499k | 35.26403 17.12585 2.06 0.045 .8483461 69.6797

500k+ | 36.13665 17.83575 2.03 0.048 .2943691 71.97893

missing | 52.54373 20.85204 2.52 0.015 10.64 94.44747

|

ibmi |

2. overwt | -23.68412 9.25317 -2.56 0.014 -42.27906 -5.08918

3. obese | -40.65356 10.22939 -3.97 0.000 -61.21028 -20.09684

|

charlsonmod | -3.828662 2.830564 -1.35 0.182 -9.516893 1.859569

|

new_adlcat |

at least 1 ADL.. | -1.102401 8.902297 -0.12 0.902 -18.99224 16.78743

|

new_iadlcat |

at least 1 IAD.. | -2.386958 7.807337 -0.31 0.761 -18.07639 13.30247

missing | -9.018964 12.59153 -0.72 0.477 -34.32259 16.28466

|

new_slowgait2 |

1 | -9.165855 8.126623 -1.13 0.265 -25.49692 7.165206

missing | 7.530202 26.43577 0.28 0.777 -45.59447 60.65487

|

new_posweak2 |

1 | -12.64809 7.221345 -1.75 0.086 -27.15993 1.863748

missing | 5.818249 15.35639 0.38 0.706 -25.04156 36.67806

|

moca | -1.0982 1.131799 -0.97 0.337 -3.372637 1.176236

cesd | .2104661 .8981123 0.23 0.816 -1.594358 2.01529

|

new_physhlth |

good | -.9243701 9.771286 -0.09 0.925 -20.5605 18.71176

very good | 16.99172 10.24343 1.66 0.104 -3.593219 37.57666

excellent | 5.220668 14.9809 0.35 0.729 -24.88458 35.32591

|

_cons | 354.5343 70.12383 5.06 0.000 213.6152 495.4534

-----------------------------------------------------------------------------------

Note: 1 stratum omitted because it contains no subpopulation members.

(running regress on estimation sample)

**Model #4: Community Involvement – Minimally Adjusted**

Survey: Linear regression

Number of strata = 49 Number of obs = 3,361

Number of PSUs = 98 Population size = 3,362.3439

Subpop. no. obs = 598

Subpop. size = 609.578668

Design df = 49

F( 15, 35) = 53.11

Prob > F = 0.0000

R-squared = 0.0856

-------------------------------------------------------------------------------

| Linearized

mean_daily_~m | Coef. Std. Err. t P>|t| [95% Conf. Interval]

--------------+----------------------------------------------------------------

cat3community | 4.504653 2.051498 2.20 0.033 .382014 8.627291

|

day1_mon |

2 | 19.89549 28.5467 0.70 0.489 -37.47126 77.26224

3 | -26.75573 24.97445 -1.07 0.289 -76.94376 23.4323

4 | 27.47202 24.5225 1.12 0.268 -21.80779 76.75182

5 | 4.589044 23.16879 0.20 0.844 -41.97038 51.14847

6 | 38.39033 40.42384 0.95 0.347 -42.84442 119.6251

8 | -110.1933 21.7414 -5.07 0.000 -153.8843 -66.50235

9 | -.7539882 23.30174 -0.03 0.974 -47.58059 46.07261

10 | 5.584329 19.79862 0.28 0.779 -34.2025 45.37116

11 | 13.39334 21.49904 0.62 0.536 -29.81059 56.59727

12 | 18.7294 23.77779 0.79 0.435 -29.05386 66.51265

999 | 280.3356 203.067 1.38 0.174 -127.7428 688.414

|

weekend_sum |

1 | -13.8965 9.094616 -1.53 0.133 -32.17281 4.379817

2 | -17.35735 9.535662 -1.82 0.075 -36.51998 1.805277

|

n_total_cpm | .0082056 .0089575 0.92 0.364 -.0097951 .0262063

_cons | 189.0702 32.20621 5.87 0.000 124.3494 253.791

-------------------------------------------------------------------------------

Note: 1 stratum omitted because it contains no subpopulation members.

(running regress on estimation sample)

**Model #4: Community Involvement – Fully Adjusted**

Survey: Linear regression

Number of strata = 49 Number of obs = 3,353

Number of PSUs = 98 Population size = 3,355.8587

Subpop. no. obs = 590

Subpop. size = 603.093484

Design df = 49

F( 45, 5) = 25.21

Prob > F = 0.0009

R-squared = 0.3346

-----------------------------------------------------------------------------------

| Linearized

mean_daily_cpm | Coef. Std. Err. t P>|t| [95% Conf. Interval]

------------------+----------------------------------------------------------------

cat3community | 3.333125 1.834972 1.82 0.075 -.3543892 7.02064

|

day1_mon |

2 | 19.41321 27.34409 0.71 0.481 -35.53679 74.36321

3 | -18.76285 19.50607 -0.96 0.341 -57.96177 20.43607

4 | 23.3012 23.74654 0.98 0.331 -24.41927 71.02167

5 | 18.586 23.46731 0.79 0.432 -28.57333 65.74532

6 | 29.92725 29.13729 1.03 0.309 -28.62633 88.48084

8 | -85.98731 29.47129 -2.92 0.005 -145.2121 -26.76254

9 | 25.38033 15.85988 1.60 0.116 -6.491287 57.25195

10 | 18.51238 15.20882 1.22 0.229 -12.0509 49.07565

11 | 20.44296 18.46468 1.11 0.274 -16.66321 57.54912

12 | 20.05753 18.38972 1.09 0.281 -16.898 57.01305

999 | 273.6013 179.805 1.52 0.135 -87.73044 634.9331

|

weekend_sum |

1 | -7.600684 8.961447 -0.85 0.400 -25.60939 10.40802

2 | -12.48616 7.618393 -1.64 0.108 -27.79589 2.823579

|

n_total_cpm | .0096579 .0073396 1.32 0.194 -.0050916 .0244075

age | -3.003833 .6500965 -4.62 0.000 -4.310251 -1.697415

|

gender |

2. female | 41.69186 8.351096 4.99 0.000 24.9097 58.47402

|

ethgrp |

2. black | -18.82284 13.01889 -1.45 0.155 -44.98528 7.33961

3. hispanic, n.. | 22.52648 14.24679 1.58 0.120 -6.103529 51.15648

4. other | 10.84273 14.77598 0.73 0.467 -18.85071 40.53617

|

educ |

2. hs/equiv | 15.06742 12.97336 1.16 0.251 -11.00353 41.13836

3. voc cert/so.. | 2.813505 12.74908 0.22 0.826 -22.80673 28.43374

4. bachelors o.. | -5.86767 17.00586 -0.35 0.732 -40.04223 28.30689

|

married |

yes | 7.153057 7.683972 0.93 0.356 -8.288462 22.59458

|

jobstat_1 |

1. yes | 18.94192 9.702805 1.95 0.057 -.5565967 38.44044

|

assets2 |

10k-49k | 18.7826 19.59624 0.96 0.343 -20.59752 58.16271

50k-99k | 31.58617 20.98425 1.51 0.139 -10.58325 73.75559

100k-499k | 39.64936 18.35543 2.16 0.036 2.762749 76.53598

500k+ | 40.56704 18.82626 2.15 0.036 2.734258 78.39982

missing | 58.26727 22.39145 2.60 0.012 13.26996 103.2646

|

ibmi |

2. overwt | -23.1747 9.575658 -2.42 0.019 -42.4177 -3.931692

3. obese | -42.38085 10.40263 -4.07 0.000 -63.28572 -21.47597

|

charlsonmod | -3.840398 2.915654 -1.32 0.194 -9.699625 2.018828

|

new_adlcat |

at least 1 ADL.. | -.7523916 9.052235 -0.08 0.934 -18.94354 17.43875

|

new_iadlcat |

at least 1 IAD.. | -2.365636 7.905395 -0.30 0.766 -18.25212 13.52085

missing | -11.46835 13.806 -0.83 0.410 -39.21255 16.27584

|

new_slowgait2 |

1 | -10.20408 8.39625 -1.22 0.230 -27.07698 6.668816

missing | 10.9457 27.34742 0.40 0.691 -44.011 65.90241

|

new_posweak2 |

1 | -15.02993 7.487672 -2.01 0.050 -30.07697 .0171099

missing | 5.480561 15.28919 0.36 0.722 -25.24422 36.20534

|

moca | -1.429044 1.154044 -1.24 0.222 -3.748183 .8900947

cesd | -.1224145 .9492402 -0.13 0.898 -2.029984 1.785155

|

new_physhlth |

good | -1.652877 10.08937 -0.16 0.871 -21.92822 18.62247

very good | 15.06894 10.54238 1.43 0.159 -6.116756 36.25464

excellent | 1.927473 15.21658 0.13 0.900 -28.65139 32.50633

|

_cons | 391.1174 77.57443 5.04 0.000 235.2258 547.0091

-----------------------------------------------------------------------------------

Note: 1 stratum omitted because it contains no subpopulation members.

(running regress on estimation sample)
